# Dexa Body Composition Assessment in 10-11 Year Healthy Children

**DOI:** 10.1371/journal.pone.0165275

**Published:** 2016-10-27

**Authors:** W. M. Verduin, R. Van Den Helder, H. J. Doodeman, E. Struijf, A. P. J. Houdijk

**Affiliations:** 1 Department of Surgery, North west Clinics, Alkmaar, The Netherlands; 2 Foreest Medical School, North West Clinics, Alkmaar, The Netherlands; 3 Municipal Public Health Care Organisation Hollands Noorden, Alkmaar, The Netherlands; University of Oslo, NORWAY

## Abstract

**Introduction:**

Obesity is a growing health problem associated with metabolic derangements and cardiovascular disease. Accumulating evidence links the accumulation of visceral adipose tissue (VAT) to these obesity related health risks in adults. Childhood obesity is associated with a lifetime risk of cardiovascular disease and poses a serious challenge to future health care. In children, there is much less data on the prevalence and gender differences of visceral obesity than in adults. This study aims to provide reference values for VAT in children 10–11 years of age.

**Methods:**

In a cross-sectional study performed in the north western part of theNetherlands, healthy children of 10–11 years of age, were recruited from primary schools. Anthropometric data consisting of height, weight, waist circumference (WC) and BMI were measured. Body composition was measured using DXA, providing measures for bone mineral content, total fat mass (TFM), lean body mass (LBM) and VAT.

**Results:**

217 children were eligible for this study. Girls appeared to have a greater TFM (31.4% vs 27.5% of total body weight (TBW); P < .01) but lower VAT (0.3% vs 0.5% of TBW;P < .01) than boys, whereas boys had higher LBM (65.4% vs 69.3% TBW;P < .01). Median VAT area (cm^2^) was 41.1 for boys and 22.4 for girls (P < .01). Moderate to strong correlations were found for WC and BMI with VAT (boys: *r* = .664 and *r* = .630; Girls *r* = .699 and *r* = .546 respectively all P < .001).

**Discussion:**

This study shows gender specific differences in VAT percentiles in healthy non-obese 10–11 year old children as measured by DXA that may serve as reference values in children. Independent of BMI and WC, girls tend to have more TFM but less VAT and LBM than boys.

## Introduction

Obesity is a growing health problem associated with the metabolic syndrome, hypertension and cardiovascular morbidity. In children and adolescents, the prevalence of overweight and obesity has shown a dramatic increase in recent years.[1, 2] Because there is a clear association between childhood obesity and lifetime risk of cardiovascular disease, this poses a serious challenge to future health care.

Increasing evidence on the cause of the metabolic derangements accompanying obesity points to the excessive accumulation of visceral adipose tissue (VAT). Visceral obesity induces a chronic inflammatory state mediated by fat-infiltrated immune cells, that in turn sets off insulin resistance, diabetes and cardiovascular disease.[3] Most studies on the associated risks between VAT and metabolic and cardiovascular disease have focussed on the adult population.[4]

In children, less is known on the accumulation and sex differences of VAT. A number of studies found boys to have greater amounts of VAT, whereas others showed more VAT in girls.[5–10] One study even found no significant differences between boys and girls.[11] The majority of these studies were performed in small sample sizes of 20–60 participants and in a broad age range. Taken together, the literature is inconclusive in regard of sex differences for VAT.[12]

In order to further elucidate the association between VAT and obesity-related comorbidities in children, there is a need for reference values from healthy non-obese boys and girls [13–17]. In the north western part of the Netherlands the childhood obesity rate is low (3.4%) and thus a suitable region for creating a body composition dataset for reference purposes.

Anthropometric measures, like waist circumference (WC) and body mass index (BMI), are used as surrogate determination of body fat. However, these measures only have a moderate correlation with the absolute and relative amount of VAT as determined by imaging techniques such as MRI, CT and DXA.[18, 19] Of these imaging techniques, DXA seems the most suitable method to measure body composition in the general population. It measures adipose tissue mass and distribution with great accuracy, accompanied by safe low radiation, short scanning time and low cost.[20, 21] By accurately measuring adipose tissue mass and its distribution, we aim to aid in determining the consequences of paediatric visceral obesity in order to target children at increased risk for future disease and allow for a timely intervention.[22]

The primary objective of this study was to create reference values for VAT in non-obese boys and girls 10–11 years of age. Secondary objectives were to investigate the differences in adipose tissue mass and lean body mass distribution between boys and girls and to investigate the associations between body composition and anthropometric measures.

## Methods

### Participants

This cross-sectional study was performed in north-western part of The Netherlands. This region is characterized by a low incidence of childhood obesity (3.4%).[23, 24] Boys and girls of 10–11 years of age were eligible and recruited from 13 primary schools, contacted through the Municipal Public Health Care Organisation Hollands Noorden. The boards of the schools were contacted and informed of the study. Schools were offered an educational day at the stadium of the local professional soccer team AZ. During that day, the children who had full consent for participation underwent a DEXA scan on the scanner that was stationed in the stadium. Participation required informed assent from the children and written informed consent by their legal representatives. This study was approved by the ethics committee of the VU University Medical Centre, Amsterdam, The Netherlands.

### Measurements

Participants’ age and gender were recorded. The anthropometric data were assessed according to Municipal Public Health Care protocol by a trained professional and consisted of the following: height as measured using a fixed wall stadiometer, total body weight in kilograms as measured with the DXA scanner, waist circumference and body mass index.[25] Body composition was analysed by means of a DXA scan (Hologic Discovery A, Advanced Body Composition^™^ assessment, with InnerCore). Total effective radiation dose was 2.6 μSv for a three minute scan.[26] DXA scanning provided values for total body weight in grams, (TBW), bone mineral content (BMC), total fat mass (TFM) and lean body mass (LBM) in grams and percentages of total body weight. Software segmentation provided a measure for VAT in square centimetres.[27] VAT measurement in square centimetres using DXA is very accurate with correlations ranging from 0.87 to 0.98 in different subgroups.[28–30] Cut-off values for BMI to determine overweight/obesity in boys were 19.8/24.0 kg/m^2^ for 10-year olds and 20.6/25.1 kg/m^2^ for 11-year olds. In girls of 10 and 11 years old, overweight/obesity cut-off values were 19.9/24.1 kg/m^2^ and 20.7/25.4 kg/m^2^, respectively. [31]

### Statistical Analyses

All variables, except for sex, were described as means with standard deviations (SD). Differences between boys and girls were tested using the independent samples T-test.

Reference values were described as 10^th^, 50^th^, and 90^th^ percentiles. Bootstrap re-sampling loops (n = 1000) were performed to construct of 95% confidence intervals for the percentiles.

Associations between anthropomorphic measures and body composition (TFM and VAT) were expressed as correlation Pearson’s coefficients and classified as no/weak correlation (0<r<0,5), moderate correlation (0,5<r<0,7) or strong correlation (0,7<r<1).

A p-values ≤0,05 was considered statistically significant. SPSS version 20 was used for statistical analyses.

## Results

### Participants

A total number of 335 children were informed, for 217 children (64.8%) informed consent was obtained from their legal representatives and participated in this study. Of these, 104 (48%) were males and 113 females. All participant characteristics are summarized in Table 1. No significant gender differences were found. In our cohort, 19 girls and eight boys were found to be overweight and one girl to be obese. All variables were normally distributed.

**Table 1:** Participants Characteristics.

	Total group(N = 217)	Male(N = 104)	Female(N = 113)	P-value
**Age**, mean (SD)	10.44 (0.5)	10.5 (0.5)	10.4 (0.5)	.056
**Weight (Kg)**, mean (SD)	39.9 (6.5)	39.3 (5.6)	40.4 (7.3)	.199
**Height (cm)**, mean (SD)	150 (6.2)	150 (5.7)	149 (6.7)	.606
**BMI (Kg/m**^**2**^**)**, mean (SD)	17.7 (2.3)	17.4 (2.2)	18.0 (2.4)	.083
**Waist circumference (cm)**, mean (SD)	62.4 (5.4)	62.8 (5.1)	62.0 (5.7)	.265

BMI, body mass index.

P-values were calculated using independent samples T-tests.

### Body Composition Results and Reference Values for VAT

Table 2 summarises all measures of body composition. Significant gender differences were observed for all DXA scan measures, except TBW. Girls had higher TFM, but lower relative VAT, than boys (both, P < .001). Boys had higher LBM and BMC than the girls (P < .001).

**Table 2:** Relative body composition.

	Total group(N = 217)	Male(N = 104)	Female(N = 113)
**TBW**, Kg (SD)	39.9 (6.5)	39.3 (5.6)	40.4 (7.3)
**BMC**^#^, % (SD)	3.1 (0.3)	3.2 (0.4)	3.1 (0.4)*
**TFM**^#^, % (SD)	29.5 (5.4)	27.5 (4.9)	31.4 (5.1)*
**LBM**^#^, % (SD)	67.3 (5.2)	69.3 (4.7)	65.5 (4.9)*
**VAT**^#^, % (SD)	0.40 (0.14)	0.5 (.10)	0.30 (.10)*

* significant at P = < .01

TBW, total body weight; BMC, bone mineral content; TFM, total fat mass; LBM, lean body mass; VAT, visceral adipose tissue.

p values were calculated using independent samples T-tests.

^#^ Percentage relative to TBW

**Fig 1:**
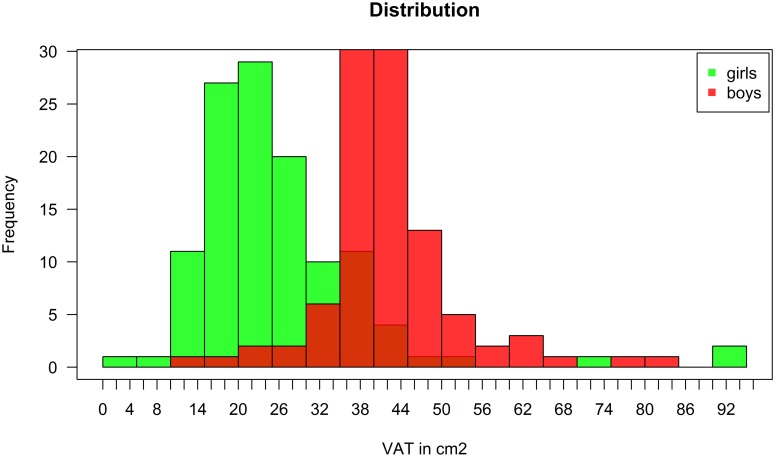
Visceral adipose tissue (VAT) distribution in girls and boys.

Table 3 depicts gender specific percentiles of VAT area in square centimetres with their respective 95% confidence intervals. VAT area is higher in boys across all percentiles and is visualized in Fig 1.

**Table 3:** Percentiles for VAT.

Percentile		5	10	25	50	75	90	95
	Total	14.3	17.5	21.6	35.5	41.6	48.2	54.0
VAT in cm^2^	Male	28.4	34.6	37.2	41.1	44.8	50.8	58.6
	Female	13.1	14.8	18.8	22.4	30.6	39.2	45.2

VAT, visceral adipose tissue

### Body Composition and Anthropometric Measures

In both boys and girls, WC and BMI showed a stronger correlation with TFM than VAT. Anthropometric measures correlated moderately with VAT in cm^2^ and strongly with TFM. The WC appears to have a stronger correlation with VAT mass, than BMI. All correlation coefficients are summarized in Table 4.

**Table 4:** Correlation coefficients for VAT and TFM with anthropometric measures.

	Coefficient	P-value
Boys		
VAT vs WC	.664	.000
TFM vs WC	.856	.000
VAT vs BMI	.630	.000
TFM vs BMI	.885	.000
Girls		
VAT vs WC	.699	.000
TFM vs WC	.817	.000
VAT vs BMI	.546	.000
TFM vs BMI	.776	.000

VAT, visceral adipose tissue; WC, waist circumference; TFM, total fat mass; BMI, body mass index.

P values were calculated using Pearson’s correlation coefficients.

## Discussion

To our knowledge, the present study provides the largest data set on DEXA body composition in 10-11-year-old non-obese healthy children. Our data are the first to show sex-specific VAT in percentiles for this age group. Schnurbein, et al. reported on reference values of VAT in millimetres as measured by ultra-sonography on a broad age group of children.[32] The use of ultra-Sonography, however, requires trained physicians and poses more difficulty for measuring VAT which in turn leads to a lower intra-observer reliability than DXA.[32] Another benefit of DXA is the relatively fast assessment of total body composition. Our data show that 10- to 11-year-old girls, although having a higher mean TFM, have a lower amount of VAT and LBM than boys of the same age with no differences in BMI or WC.

Staiano, et al. studied children within a broad age range from 5–18 years and also reported more TFM in girls and higher VAT in boys.[33] However no sex differences in VAT area before the age of 15 were noted. Satake, et al. reported similar findings in 130 non-obese Japanese children.[34] They divided the children in 3 age groups ranging from 6 through 20 years of age. They noticed more VAT in boys only in the highest age group of 16–20 year olds. Our study shows that in non-obese children, these sex differences are already present at the age of 10–11.[33, 34]

In overweight and obese children in a broad age range with a median of 11.5 years, Druet, et al. reported no differences in TFM between boys and girls with boys having higher VAT.[13] This suggests that the difference in TFM we found between boys and girls of normal weight may disappear with an increase in bodyweight. Because the weight range is this study is to small and only 0.5% of our group was obese, we cannot substantiate this. More studies in overweight and obese children using DXA are needed.

In contrast to our study, Fox, et al. found no significant sex differences in VAT in a sample of 50 boys and girls 11 years of age using MRI.[35] In addition, a study by Gowers, et al. in 38 children also found no significant differences in VAT in cm^2^ as measured by CT.[36]

Possible explanations for some of the contradictory results might be related to the smaller sample size of these studies, broad age ranges and the use of different imaging techniques.

Percentiles are widely used in paediatric growth and development monitoring. For the first time, we show reference values in percentiles for body composition in healthy non-obese 10–11 year old children as measured by DXA. These reference values for VAT in young children may help to detect those who are at risk for developing the metabolic syndrome with advancing age. Our study was executed in a Dutch region characterized by a low incidence of overweight (16%) and obesity (3.4%).[23, 24] The incidence for overweight in our study was 12.4% and one girl was obese (0.5%). Our cohort therefore consisted of non-obese, healthy subjects and our data may be considered representative for values dating from before the current obesity epidemic. Retrospective analysis of the standard error of the 90^th^ percentiles calculated after each tenth inclusion shows that the inclusion of more participants would not have changed the outcome substantially.

In comparison to the literature on VAT area and signs of the metabolic syndrome, our data indeed may be used as reference values for healthy children. Asayama, et al. studied 290 obese Japanese children in the age range of 6 through 15 years for abnormalities in serum triglyceride, alanine aminotransferase or fasting insulin levels. They found a critical VAT area of 54.8 cm^2^ for the occurrence of abnormal values corresponding to the 95th percentile of 54.0 cm^2^ in the present study.[8] Weiss, et al. studied insulin resistance in 28 obese American adolescents with a mean age of 13.8. They matched the groups in an insulin sensitive and insulin insensitive group. In the insulin sensitive group, they found a mean VAT area of 45 cm^2^ and 76 cm^2^ in the insulin insensitive group.[37] Considering these data, the VAT percentiles in the present study all fall into the healthy range and therefore can be used as reference values for the non-obese 10–11 year old child.

A strong point of our study is the use of DXA for measuring body composition. DXA is a very reliable imaging technique, with little inter- and intra-observer variability. It can be safely used for repeated routine use in children and adults, because of the non-invasive character and the low dose of ionizing radiation.[21] The accuracy of measuring body composition with DXA stresses the advantage of using DXA over BMI or WC in accurately assessing body composition while maintaining an approach that remains feasible in routine daily practice in comparison to CT or MRI. Although scanning requires children to lie still for three to four minutes with proper instructions, we found that our 10- to 11-year-old participants cooperated well, resulting in high quality imaging and analyses. Taking these statements into account, we believe body composition imaging, especially DXA, can be safely, practically and efficiently performed for routine clinical growth- and development monitoring in this age group.

Furthermore, data were collected during an event that promoted healthy lifestyle for primary school children. With this approach we were able to combine data collection from participating primary schools. By intertwining cross-sectional research with an educational event, we believe consent to participation was encouraged, while saving costs.

Our study has several limitations, challenging future studies. The current availability of out-of-hospital DXA scanning is scarce, limiting our study sample to one Dutch region. In addition, our group represents healthy lean children of a single ethnicity that may not represent the general Dutch multicultural population of 10- to 11-year-olds. Body composition in adults and obesity or obesity-related diseases in adults and children are known to vary within the Netherlands due to genetic and cultural variances. Furthermore, because of ethical concerns and in order to warrant feasibility by using DXA during a public health event, maturity level was not assessed. Maturity obviously plays a major role in determining body composition, with adipose tissue and muscle development being hallmarks of puberty with great gender differences. Our age group of 10–11 years is considered mainly pre-pubertal.[38] Another possible limitation is related to the fact that only 64.8% of the children had written informed consent. It might be possible for instance, that among the children who were not scanned, more children had obesity than in our study sample. However, at the low obesity rate in the region and in our study this possible bias is of limited importance. Furthermore, our inclusion rate actually was quite high considering the fact that recruitment rates reported for children studies range from 18.5% through 58.5%.[39] Personally contacting the schools and combining the study days with an educational event most likely explain this high recruitment rate.

In conclusion, the VAST study provides the largest data set on DEXA body composition in healthy non-obese 10–11 year old Dutch children that may be used for reference purposes in this age group. Girls tend to have higher total fat mass and boys have more VAT. This is of major importance because in determining adiposity-related health, VAT is now considered a principal etiologic factor.

With respect to variation due to age, sex, lifestyle and ethnicity, further research should include larger cohorts and address implementation of VAT measurement in routine growth and development monitoring.

## Supporting Information

S1 Database (SAV)
